# Magnetic Nanobeads as Support for Zinc(II)–Cyclen Complexes: Selective and Reversible Extraction of Riboflavin

**DOI:** 10.1002/open.201200008

**Published:** 2012-06-11

**Authors:** Quirin M Kainz, Andreas Späth, Stefan Weiss, Thomas D Michl, Alexander Schätz, Wendelin J Stark, Burkhard König, Oliver Reiser

**Affiliations:** a Institut für Organische Chemie, Universität Regensburg, Universitätsstr. 3193053 Regensburg (Germany) E-mail: Burkhard.Koenig@chemie.uni-regensburg.deOliver.Reiser@chemie.uni-regensburg.de; b Institute for Chemical and Bioengineering, Swiss Federal Institute of Technology (ETH) ZurichHCI E 107, Wolfgang-Pauli-Str. 10, 8093 Zurich (Switzerland)

**Keywords:** chelates, fluorescence spectroscopy, molecular recognition, nanoparticles, polymerization

Riboflavin (vitamin B_2_) is essential for the health of humans and animals. Its structure is part of flavin adenine dinucleotide (FAD) and riboflavin-5′-phosphate (flavin mononucleotide, FMN), redox cofactors found among others in dehydrogenases, oxidases and photolyases.[1] A lack of vitamin B_2_ can cause growth failure, seborrheic dermatitis or even cataracts and normocytic anemia.[2] Therefore, extraction and quantification of riboflavin in body fluids, like urine or blood plasma, or in nutrition supplements, for example vitamin tablets, is of analytical interest.[3]

It has been shown that artificial receptors containing zinc(II)–cyclen complexes can reversibly coordinate imide moieties, like thymine, uracil, and flavins.[4] Recent reviews outline the molecular interactions of zinc(II)–cyclen and its derivatives.[5] We previously reported on a zinc(II)–cyclen-functionalized polymer that reversibly and selectively binds flavins at physiological pH and enables the extraction of riboflavin from aqueous solutions.[6] A magnetic support would further improve this concept by allowing rapid agitation and separation by applying an external magnetic field. Various surface-modified magnetic nanoparticles[7] have been successfully employed for the extraction of histidine-tagged proteins,[8] DNA/RNA,[9] biotin-labeled antibodies,[10] or heavy metal ions[11] from aqueous media. We report here the immobilization of zinc(II)–cyclen complexes as binding motifs for the extraction and release of riboflavin from aqueous media on carbon-coated magnetic metal nanoparticles, featuring both high thermal and chemical stability, as well as high magnetization levels (158 emu g^−1^).[12] This concept may be applied to the extraction of other target molecules by an exchange of the receptor molecules on the surface of the magnetic nanobeads. Previously, these nanobeads have been applied as supports for catalysts[13, 14] or as magnetic scavengers.[15]

The synthesis of cyclen-functionalized nanobeads started from threefold *tert-*butoxycarbonyl (Boc)-protected cyclen **1**.[16] Propargylation applying standard conditions afforded **2** in good yields (Scheme 1). Polymer-coated magnetic support **3** was prepared by in situ grafting polymerization of 4-chloromethyl styrene on vinyl-functionalized Fe/C nanobeads following literature procedures.[17] A high loading of benzyl chloride functional groups (2.5 mmol g^−1^) was determined for polymer-encapsulated nanobeads **3** by elemental microanalysis. Despite their high loading, nanobeads **3** reach magnetization levels (33 emu g^−1^) that are typical for low-loading magnetite particles (∼1 mmol g^−1^).[18]

**Scheme 1:**
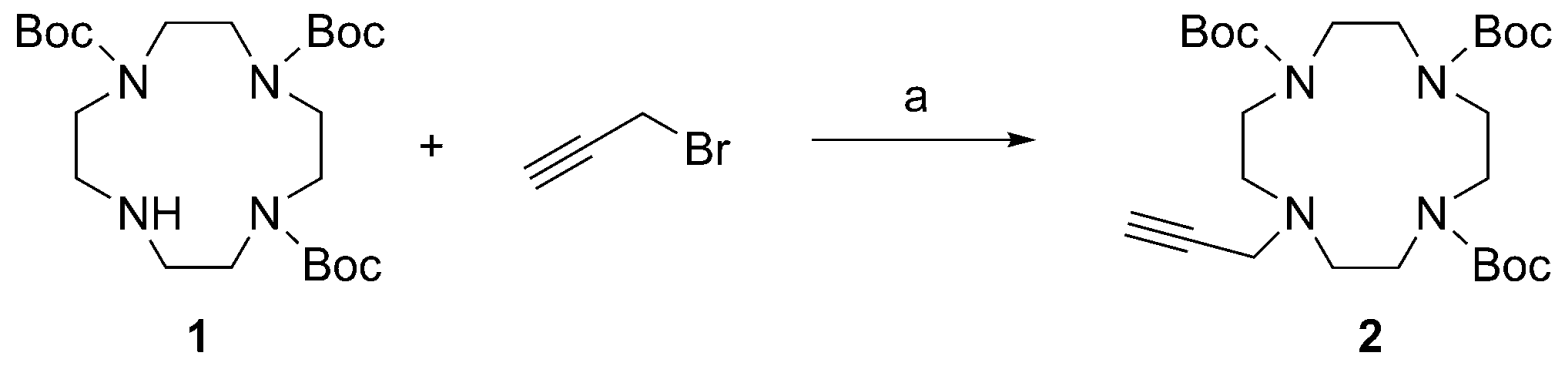
Synthesis of the propargylated and Boc-protected cyclen building block. *Reagents and conditions:* a) K_2_CO_3_, KI, MeCN, 50 °C, 4 h, 88 %.

Exchange of chloride for azide in the polymer backbone of **3** yielded magnetic beads **4** being suitable for a “click”-reaction with propargylated cyclen **2** (Scheme 2). The reaction was monitored using attenuated total reflectance (ATR) IR spectroscopy. During the course of the reaction the benzyl chloride peak at 1263 cm^−1^ decreased and a new peak at 2093 cm^−1^ was detected corresponding to the introduced azide groups.[19] The azide loading calculated from nitrogen elemental analysis was 2.0 mmol g^−1^ (83 % substitution). Subsequently, propargylated cyclen **2** was immobilized on **4** using copper-catalyzed azide–alkyne cycloaddition (CuAAC; Scheme 2).[20] To obtain a high degree of functionalization, 10 mol % copper(I) iodide was used and the nanoparticles were stirred for three days utilizing their intrinsic magnetic properties. Traces of copper and iron were removed by washing the particles with an ethylenediaminetetraacetic acid (EDTA) solution to prevent the formation of unwanted metal–cyclen complexes upon Boc deprotection in the later course of the synthesis. The completion of the reaction was conveniently monitored by IR-ATR spectroscopy, observing the decreasing azide peak at 2100 cm^−1^ and the increasing peak of the carbonyl stretch at 1677 cm^−1^.[19] Based on the molar mass of protected cyclen **2** and the initial azide loading of particles **4**, the maximum theoretical loading for the resulting nanoparticles (**5**) functionalized with protected cyclen was calculated to be 1.0 mmol g^−1^. In practice, owing to the high steric demand of **2** accompanied by limited penetration of the polymer shell, 0.67 mmol g^−1^ cyclen could be realized using CuAAC (67 % functionalization). The cyclen loading could not be further improved by a second run. Nevertheless, this cyclen loading is about five times higher than loadings in comparable studies where conventional polymer beads were used.[6]

**Scheme 2:**
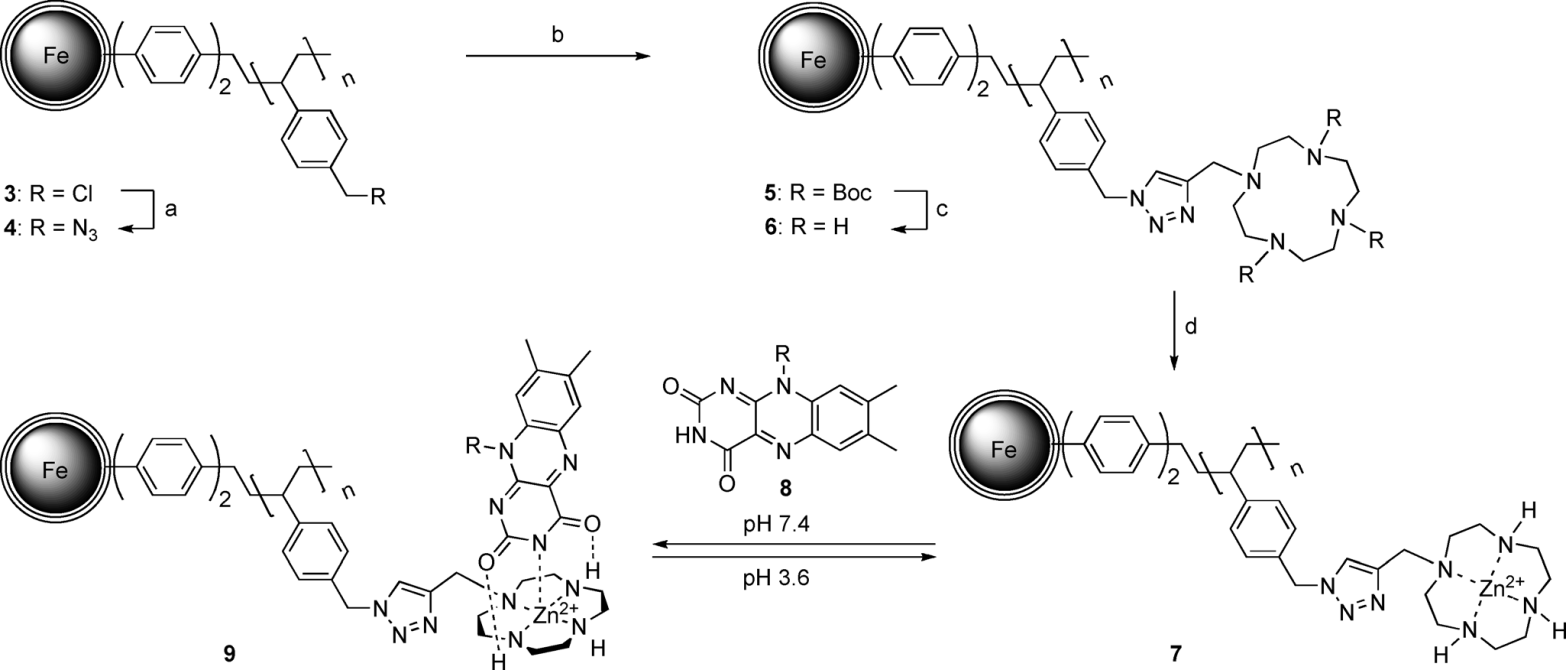
Functionalization of polymer-coated Fe/C nanoparticles with a cyclen ligand using click reaction and subsequent complexation with zinc(II). Riboflavin is reversibly coordinated by these immobilized complexes. *Reagents and conditions:* a) NaN_3_, tetrahydrofuran (THF)/H_2_O, 80 °C, 3 d; b) Boc-protected cyclen 2, CuI (10 mol %), NEt_3_, CH_2_Cl_2_, RT, 3 d; c) TFA (25 %), CH_2_Cl_2_, RT, 6 h; then NaOH (1m), 1 h; d) Zn(ClO_4_)_2_⋅6H_2_O, NaHCO_3_, H_2_O, 80 °C, 18 h.

Deprotection of **5** by treatment with trifluoroacetic acid (TFA) in dichloromethane followed by deprotonation with aqueous sodium hydroxide gave polymer-coated Fe/C nanoparticles functionalized with free cyclen ligand **6**. Subsequently, the corresponding immobilized zinc(II)–cyclen complex **7** was generated by stirring **6** with zinc(II) perchlorate hexahydrate in water at pH 8. Heating to 80 °C was sufficient for this ligand to overcome the thermodynamic barrier of the complexation process.[21] Despite the high loading with polymer and the additional functionalization with cyclen and zinc(II), **7** was readily collected with a magnet within seconds. IR-ATR investigations revealed almost complete disappearance of the carbonyl peak and the appearance of a new strong peak at 1076 cm^−1^.[19] Inductively coupled plasma atomic emission spectroscopy (ICP-AES) was used to determine the amount of zinc(II) in **7**. The measured value of 0.65 mmol g^−1^ zinc(II) is in good agreement with the value of 0.67 mmol g^−1^ cyclen determined by elemental analysis for **5**, while the residual amount of copper was negligible (0.2 μmol g^−1^). Transmission electron microscopy (TEM) analysis of **3** and **7** showed no substantial differences, indicating the successful and selective complexation, as zinc(II) ions coordinated by cyclen should not be visible by this method in contrast to unselective deposition of zinc aggregates onto the nanoparticles.[19]

In order to determine the maximum loading of **7** with riboflavin (**8**), the nanoparticles were added to a stock solution of **8**. A concentration of 0.16 mmol g^−1^ could be extracted by the nanobeads as determined by UV/Vis and fluorescence measurements,[19] which is considerably lower than the calculated value if complexation of all zinc(II)–cyclen moieties would have occurred (0.52 mmol g^−1^). It appears that not all of the binding sites can be accessed by the guest, most likely due to steric reasons.

Next, the potential application of nanocarriers **7** for the quantitative extraction of riboflavin from aqueous solutions and the recovery of riboflavin from the nanobeads by acidification was examined.[22] The typical absorption spectrum of flavin in buffer (10 μm HEPES, pH 7.4)[23] disappeared entirely after stirring with a threefold excess of nanoparticles for 5 min indicating quantitative extraction (Figure 1 a). By removing the particles via magnetic decantation and washing them with dilute hydrochloric acid (pH 3.6), almost all of the initial absorption was restored. These results were further reinforced by fluorescence measurements, also revealing full recovery of the fluorescence intensity and therefore perfect reversibility (Figure 1 b). To ensure that the observed binding is not only caused by unspecific adsorption to the polymer-coated particles, a control experiment was conducted using unmodified polymer-coated nanoparticles **3**. No binding was detected by UV/Vis or fluorescence measurements.[19]

**Figure 1:**
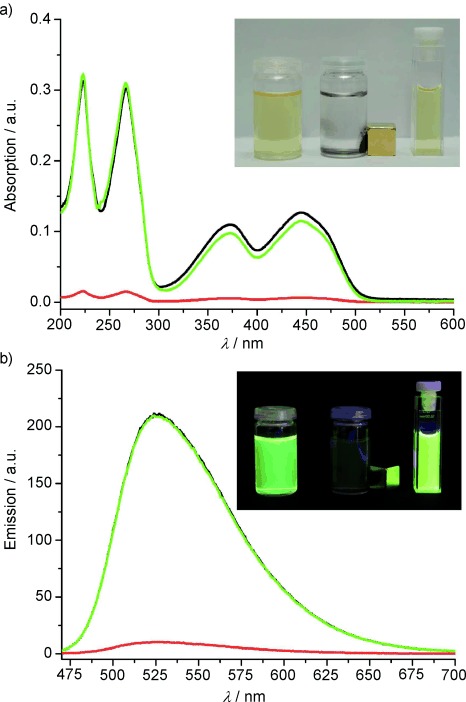
Quantitative and reversible extraction of riboflavin using polymer-coated nanobeads 7. a) Absorption spectra and b) emission spectra. Solution of riboflavin (10 μm, —), solution after treatment with 7 (—), recovery of riboflavin from 7 (—). The insets show photographs taken at normal daylight (a) and under UV light (b). The golden cube is a neodymium-based magnet.

To address the question whether a polymer is necessary to shield the nanoparticle surface from unspecific adsorption of riboflavin, or if simple functionalization with the zinc(II)–cyclen complexes is sufficient, magnetic carbon-coated cobalt nanoparticles (Co/C) bearing azide groups at the surface were prepared according to literature procedures (0.1 mmol g^−1^ azide).[13, 24] The Co/C nanobeads are identical to the previously described Fe/C nanobeads in magnetization, stability and functionalization. Cyclen **2** was immobilized via CuAAC, followed by its deprotection and complexation using similar conditions as for the synthesis of polymer-coated nanobeads **7**.[19] The amount of ligand immobilized on the surface was determined by elemental microanalysis as 0.06 mmol g^−1^. The amount of zinc absorbed was again measured by ICP-AES. However, in this case, the observed zinc-loading was 0.38 mmol g^−1^, a value about six times higher than expected for complete complexation. This indicates considerable unspecific absorption of zinc(II) onto the nanoparticles despite the functionalization with zinc(II)–cyclen complexes.

Nevertheless, we examined the potential of the non-polymer-coated nanocarriers in the extraction and recovery of riboflavin from aqueous solutions. Although the particles were added in a sixfold excess, they were not able to remove riboflavin quantitatively from the sample solution. Moreover, not all bound riboflavin can be subsequently recovered by acidification. A control experiment using unfunctionalized Co/C nanoparticles indeed proved that particles lacking the polymer, irreversibly bind considerable amounts of riboflavin to the carbon surface.[19]

The reusability of polymer-coated iron nanoparticles **7** with immobilized zinc(II)–cyclen complex was investigated by repeating the binding and release process six times using a fresh aliquot of riboflavin in buffer for each cycle (Figure 2). The binding and release ability only slightly decreased with the number of cycles, presumably by minor loss of particles in each cycle. ICP-AES measurements of the recovered nanoparticles revealed almost complete retention of zinc loading after six cycles (0.64 mmol g^−1^, >98 %), underlining the good reusability of **7** despite repetitive washing with aqueous hydrochloric acid (pH 3.6).

**Figure 2:**
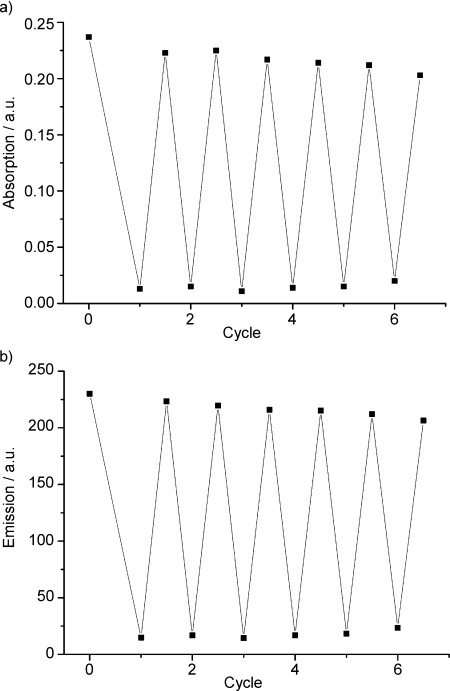
Recycling experiments using polymer-coated and zinc(II)–cyclen-functionalized nanobeads 7 (20 mg) and riboflavin-containing buffer (50 mL, 20 μm). a) Absorption of riboflavin in solution at 445 nm and b) emission at 524 nm.

Additionally, we conducted quantitative analysis of riboflavin in vitamin tablets containing various vitamins as well as other additives.[19] The tablet was dissolved in aqueous sodium hydroxide, and the solution was adjusted to pH 8. In the absorption spectrum of the vitamin-tablet solution (Figure 3), the characteristic peaks of riboflavin can be clearly identified. After treatment with **7**, these absorption bands disappear, indicating that contained flavin is completely bound to the magnetic nanoparticles. After subsequent washing of the isolated particles with aqueous hydrochloric acid (pH 3.6) and analysis of its absorption spectrum, only the typical peaks of riboflavin can be recognized in the spectrum of the washing solution. This result suggests that, of the variety of the vitamins and other substances in the tablet, only flavin was bound to the immobilized zinc(II)–cyclen complexes. The quantitative evaluation of the absorption spectrum of the washing solution resulted in a riboflavin content of 1.5 mg per tablet, which is in good accordance with the manufacturer’s specifications (1.4 mg per tablet).

**Figure 3:**
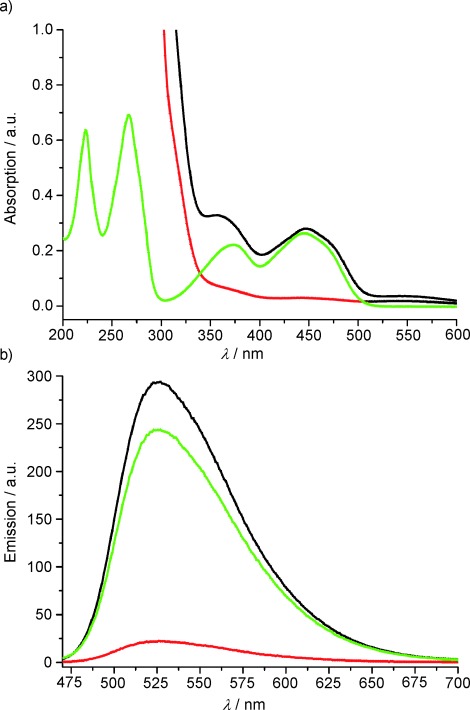
a) Absorption and b) emission spectra of riboflavin extracted from a vitamin tablet solution by zinc(II)–cyclen-functionalized nanoparticles 7. Vitamin-tablet solution (20 μm riboflavin, —), solution after treatment with 7 (—), recovery of riboflavin from 7 (—).

In conclusion, we demonstrated the successful functionalization of polymer-coated Fe/C nanobeads with zinc(II)–cyclen complexes. These nanocarriers were then applied for quantitative and reversible extraction of riboflavin from aqueous solutions. Additional experiments revealed that the polymer coating is essential to block the carbon surface of the nanobeads from unspecific adsorption of riboflavin, while simple covalent functionalization of the nanoparticles is not sufficient. The reusability of the nanobeads at high efficacy was demonstrated for six consecutive cycles. Moreover, the quantitative extraction of riboflavin from more complex matrices, for example a vitamin tablet, was also achieved. This concept could be further expanded to other important natural products by exchanging the zinc(II)–cyclen moieties on the surface of the magnetic nanobeads with other receptor molecules. Such studies are currently ongoing in our laboratories.

## Experimental Section

Experimental procedures are available in the Supporting Information.

## Supplementary material

Detailed facts of importance to specialist readers are published as ”Supporting Information”. Such documents are peer-reviewed, but not copy-edited or typeset. They are made available as submitted by the authors.
