# Supplementary material for: Magnetic Nanobeads as Support for Zinc(II)–Cyclen Complexes: Selective and Reversible Extraction of Riboflavin
Source: ChemistryOpen. 2012 Jun 11;1(3):125–9. doi: 10.1002/open.201200008 (PMC3922449; doi:10.1002/open.201200008)
Supplement: Supplementary file 1 [file open0001-0125-SD1.pdf]

ChemistryOPEN

Supporting Information

© Copyright Wiley-VCH Verlag GmbH & Co. KGaA, 69451 Weinheim, 2012

Magnetic Nanobeads as Support for Zinc(II)–Cyclen Complexes: Selective and Reversible Extraction of Riboflavin

Quirin M. Kainz,[a] Andreas Späth,[a] Stefan Weiss,[a] Thomas D. Michl,[b] Alexander Schätz,[b] Wendelin J. Stark,[b] Burkhard König,*[a] and Oliver Reiser*[a]

open_201200008_sm_miscellaneous_information.pdf

Supporting Information

1. Materials and methods S2
2. Nomenclature S2
3. Synthesis of Boc-protected cyclen S3
4. Preparation of polymer coated Fe/C nanobeads bearing zinc(II)-cyclen complexes S4
5. Functionalization of Co/C nanoparticles with zinc(II)-cyclen complexes S6
6. General procedure for the reversible extraction of riboflavin S8
7. NMR spectra S8
8. IR-ATR spectra S10
9. TEM pictures S11
10. Additional UV- and fluorescence-spectra, binding studies S11
11. References S13

1. Materials and methods

Analytical characterization of the synthesized compounds was performed by common methods. IR Spectra were recorded with a Bio-Rad FT-IR Excalibur FTS 3000 equipped with a Specac *Golden Gate* Diamond Single Reflection ATR-System. Absorption spectra were recorded on a Varian Cary BIO 50 UV/VIS/NIR spectrometer with temperature control using 1 cm quartz cuvettes (Hellma) and Uvasol solvents (Merck, Baker or Acros). Fluorescence measurements were performed with UV-grade solvents (Baker or Merck) in 1 cm quartz cuvettes (Hellma) and recorded on a Varian 'Cary Eclipse' fluorescence spectrophotometer with temperature control. Electro spray mass spectra were performed on a Finnigan MAT TSQ 7000 ESI-spectrometer. NMR spectra were recorded in $CDCl_3$ on Bruker Avance 300 (^1H: 300.1 MHz, ^{13}C: 75.5 MHz, T = 300 K) relative to external standards. Characterization of the signals: s = singlet, m = multiplet. Integration is determined as the relative number of atoms, the coupling constants are given in Hertz [Hz].
The carbon coated cobalt and iron nanomagnets (Co/C, Fe/C, 20.5 m^2/g, mean particle size ≈ 25 nm) were purchased from Turbobeads Llc, Switzerland. Prior to use, they were washed in a concentrated HCl / water mixture (1:1) 5 times for 24 h. Acid residuals were removed by washing with millipore water (5x) and the particles were dried at 50°C in a vacuum oven.[1] The magnetic nanobeads were dispersed using an ultrasound bath and recovered with the aid of a neodymium based magnet (15 x 30 mm). They were characterized by IR-ATR spectroscopy, elemental microanalysis (LECO CHN-900), transmission electron microscopy (Zeiss, LEO912AB, 100 kV) and ICP-AES (Spectro Analytical Instruments ICP Modula EOP). Poly(benzylchloride)styrene coated iron nanoparticles **3**[2] and azide functionalized cobalt nanoparticles **10**[3] and were prepared on the gram scale following previously reported procedures.
Cyclen-Boc_3 **1** was purchased from PolyPeptide Group. The vitamin pill (4.0 g) with orange flavor of TIP contains besides 1.4 mg riboflavin: Vitamin B1 (1.1 mg), vitamin B6 (1.4 mg), niacin (16 mg), folic acid (0.2 mg), Vitamin B12 (2.25 micrograms), biotin (0.05 mg), vitamin C (80 mg), vitamin E (12 mg), pantothenic acid (6.0 mg), carbohydrates (100 mg). Other ingredients are: citric acid, fructose, starch, sodium bicarbonate, dye from beetroot, sodium cyclamate and sodium saccharin. All other solvents and chemicals were of reagent grade and were used without further purification.

2. Nomenclature

The Nomenclature of the cyclen derivatives described in this work is as follows: Cyclen-Boc_3 [Tris(tert-butyl oxycarbonyl) 1, 4, 7, 10-tetraazacyclododecane] describes a cyclen azamacro-cycle with three *tert*-butoxycarbonyl protecting groups. Acet-Cyclen-Boc_3 describes a derivative bearing an acetylene moiety.
For the nanoparticles the nomenclature is as follows: Co/C for magnetic nanoparticles with cobalt core and carbon shell and Fe/C for iron nanoparticles with carbon shell. Fe/C-PS-R for polystyrene coated iron nanoparticles, where R indicates the functional groups in the polymeric

network: Cl for benzylchloride, N_3 for benzylazide, Cyclen-Boc_3 for *tert*-butoxycarbonyl protected cyclen, Cyclen for unprotected cyclen, and Cyclen-Zn for cyclen coordinating zinc(II).

3. Synthesis of Boc-protected cyclen

*Acet-Cyclen-Boc_3 (**2**):*[4]

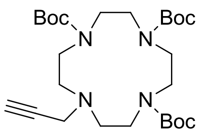

Cyclen-Boc_3 **1** (940 mg, 2.0 mmol) was dissolved in acetonitrile (10 ml), propargylbromide (80% in toluene, 484 mg, 0.4 mL), potassium carbonate (414 mg, 3.0 mmol) and potassium iodide (333 mg, 2.0 mmol) were added. The suspension was stirred for 2 h at room temperature and was then heated to 50°C for 4 h under light protection and nitrogen atmosphere. After cooling to room temperature a second portion of propargylbromide (80% in toluene, 484 mg, 0.4 mL) was added and the reaction mixture was kept at 50°C under light protection and nitrogen atmosphere for 30 h. After cooling to room temperature ethyl acetate (20 mL) was added, the mixture was filtered over celite and the filter cake was washed with small amounts of ethyl acetate. The solvent was evaporated and the residue was purified by column chromatography with ethyl acetate / hexanes (1:2) to yield the product as colorless solid (900 mg, 1.76 mmol, 88%).

Mp: 103 - 105 °C; **^{1}H-NMR** (300 MHz, $CDCl_3$): δ [ppm] = 3.55 – 3.09 (m, 14 H), 2.78 – 2.60 (m, 4 H), 2.13 (s, 1 H), 1.39 (s, 27 H); **^{13}C-NMR** (75 MHz, $CDCl_3$, signal doubling due to rotamers): δ [ppm] = 155.99, 155.70, 155.25, 79.68, 79.45, 79.24, 73.68, 54.31, 53.19, 49.91, 47.86, 47.68, 47.05, 46.53, 39.08, 28.75, 28.49; **IR** (KBr): $\bar{\nu}$ [cm^{-1}] = 3251, 2976, 2932, 2834, 1674, 1461, 1457, 1414, 1402, 1364, 1248, 1152, 1104, 1031, 980, 946, 917, 859, 772, 730, 647; **MS** (ESI-MS): m/z (%) = 511.2 (100, MH^+), calc. 510.34.

4. Preparation of polymer coated Fe/C nanobeads bearing zinc(II)-cyclen complexes

*Azide functionalized polymer coated iron nanoparticles (Fe/C-PS-N_3, **4**):*

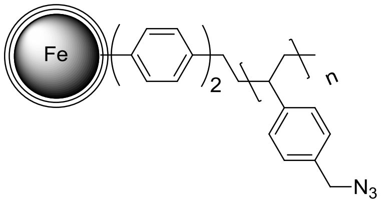

Poly(benzylchloride)styrene coated Fe/C nanoparticles **3** (1 g, 2.49 mmol) were dispersed in 10 mL of a THF / deionized water (Millipore) mixture (1:1) using an ultrasonic bath, 1.62 g (24.9 mmol) of sodium azide was added and the resulting slurry heated to 80 °C for 3 d. The particles were recovered by the aid of a magnet and washed with a THF / deionized water (Millipore) mixture (1:1, 5 x 10 mL) and acetone (3 x 10 mL). After evaporation of the solvent, 1.03 g of Fe/C-PS-N_3 were obtained.
IR (ν/cm^{-1}): 2923, 2093, 1510, 1264, 813, 670; **elemental microanalysis** (%): C, 32.85; H, 2.49; N, 8.56; Cl, 2.83.

*"Click"-reaction to generate polymer coated iron nanoparticles functionalized with Boc-protected cyclen (Fe/C-PS-Cyclen-Boc_3, **5**):*

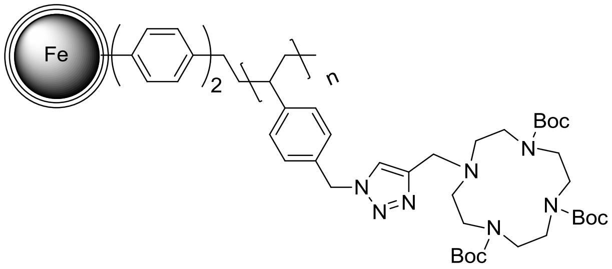

To 400 mg (0.82 mmol) of Fe/C-PS-N_3 dispersed in 10 mL of degassed dichloromethane 1.27 g (2.46 mmol) of Acet-Cyclen-Boc_3, 29 mg (0.15 mmol) of copper(I) iodide and 208 µL (1.5 mmol) triethylamine were added. The reaction mixture was stirred at room temperature for 3 d. Then, the particles were collected by a magnet and washed with dichloromethane (5 x 5 mL), aqueous EDTA (3 x 5 mL), water (3 x 5 mL) and acetone (3 x 5 mL). After drying in vacuum 805 mg of Fe/C-PS-Cyclen-Boc_3 were obtained.
IR (ν/cm^{-1}): 2974, 2932, 1677, 1456, 1413, 1364, 1247, 1151, 942, 857, 822, 722, 731, 700; **elemental microanalysis** (%): C, 43.36; H, 5.55; N, 8.01; Cl, 2.45.

*Deprotection of the Fe/C-PS-Cyclen-Boc$_3$ to the corresponding free cyclen (Fe/C-PS-Cyclen, **6**):*

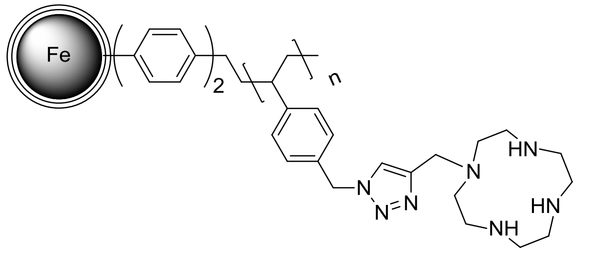

Fe/C-PS-Cyclen-Boc$_3$ nanoparticles **5** (700 mg) were stirred in 10 mL of a trifluoroacetic acid / dichloromethane mixture (1:3) at ambient temperature for 6 h. After magnetic decantation the particles were washed with dichloromethane (3 x 10 mL). Deprotonation of the cyclen moieties was achieved by stirring the particles in 10 mL of aqueous NaOH (1M) for 3 h. Finally, the particles were collected by the aid of a magnet and washed with water (3 x 5 mL) and dichloromethane (5 x 10 mL). After evaporation 450 mg of Fe/C-PS-Cyclen **6** were yielded.
IR (ν/cm^{-1}): 3252, 2917, 2811, 1685, 1510, 1439, 1345, 1196, 1110, 1042, 912, 782; **elemental microanalysis** (%): C, 38.04; H, 4.76; N, 10.73; Cl, 0.73.

*Synthesis of zinc(II)-cyclen complexes at the surface of magnetic nanoparticles (Fe/C-PS-Cyclen-Zn, **7**):*

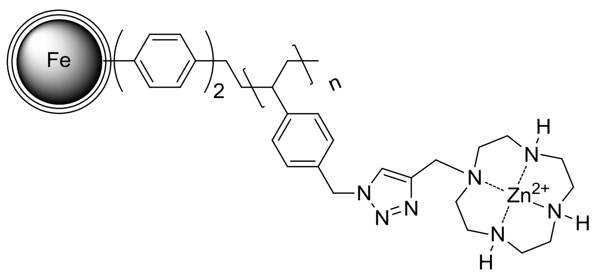

A suspension of 370 mg of Fe/C-PS-Cyclen **6** and 2.48 g (6.66 mmol) of $Zn(ClO_4)_2 \cdot 6\ H_2O$ in 10 mL of deionized water (Millipore) was prepared. The reaction mixture was buffered to pH 8 by the addition of $NaHCO_3$ and stirred at 80 °C for 18 h. After cooling down to room temperature, the nanoparticles were recovered by a magnet and washed with water, methanol and acetone (5 x 5 mL each). 422 mg of Fe/C-PS-Cyclen-Zn **7** were obtained after drying.
IR (ν/cm^{-1}): 3278, 2918, 2871, 1700, 1439, 1355, 1220 1076, 928, 788; **elemental microanalysis** (%): C, 33.26; H, 4.04; N, 9.23; Cl, 4.16; **ICP-AES**, Zn: 0.65 mmol/g.

5. Functionalization of Co/C nanoparticles with zinc(II)-cyclen complexes

Scheme S1. Synthesis of zinc(II)-cyclen functionalized Co/C nanobeads.

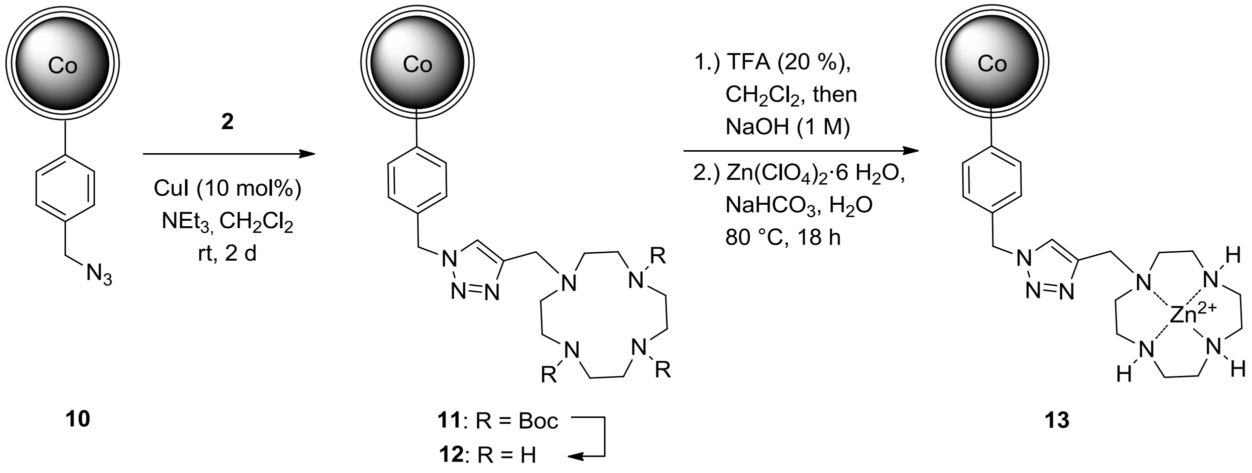

Synthesis of cobalt nanoparticles functionalized with Boc-protected cyclen (Co/C-Cyclen-Boc$_3$, ***11****):*

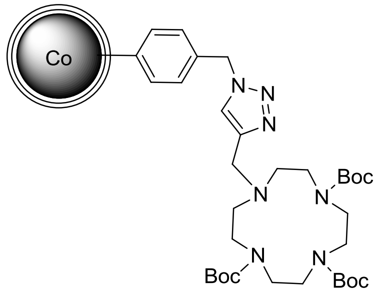

Azide functionalized Co/C nanoparticles **10** (200 mg, 0.02 mmol) were pre-dispersed in 5 mL dichloromethane by the aid of an ultrasonic bath. Thereafter, 51 mg of Acet-Cyclen-Boc$_3$ (0.1 mmol), 2 mg of CuI (0.01 mmol) and 14 µL of triethylamine (0.1 mmol) were added and the sonication continued for 1 h followed by stirring for 2 d at room temperature. The particles were collected by applying a magnet to the side of the reaction vessel and the supernatant was decanted. The particles were then washed with dichloromethane (5 x 5 mL), aqueous EDTA (3 x 5 mL), water (3 x 5 mL) and acetone (3 x 5 mL). After evaporation of the solvent, 201 mg of Co/C-Cyclen-Boc$_3$ **11** nanoparticles were obtained.
IR (ν/cm^{-1}): 2980, 2927, 1687, 1600, 1458, 1417, 1367, 1251, 1175, 1108, 1016, 785; **elemental microanalysis** (%): C, 8.7; H, 0.24; N, 0.68.

*Generation of the free cyclen tethered to the surface of magnetic cobalt nanoparticles (Co/C-Cyclen, **12**):*

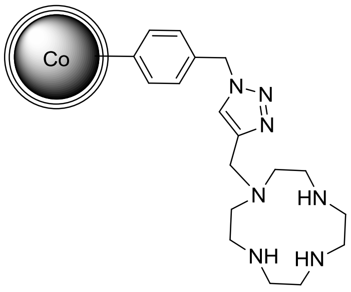

Boc-protected particles **11** (130 mg) were stirred in a trifluoroacetic acid / dichloromethane mixture (1:4) for 3 h. After magnetic decantation and washing of the nanobeads with dichloromethane (3 x 5 mL), 5 mL of aqueous NaOH (1 M) were added and the stirring continued for 3 h. The particles were collected by a magnet and again washed with water (5 x 5 mL) and dichloromethane (5 x 5 mL). Drying under vacuum yielded 122 mg of Co/C-Cyclen nanobeads.
IR (ν/cm^{-1}): 2917, 1647, 1593, 1381, 1217, 1011, 820; **elemental microanalysis** (%): C, 8.76; H, 0.18; N, 0.63.

*Complexation of the free cyclen at the surface of magnetic cobalt nanoparticles with zinc(II) (Co/C-Cyclen-Zn, **13**):*

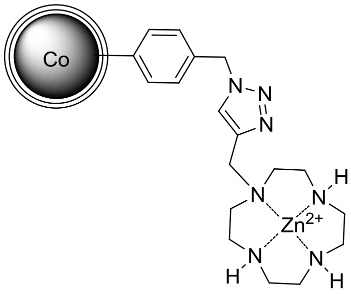

Nanoparticles **12** (100 mg) were dispersed in 5 mL water by sonication followed by addition of 45 mg (0.12 mmol) $Zn(ClO_4)_2 \cdot 6\ H_2O$ and $NaHCO_3$ to buffer the solution to pH 8. The slurry was stirred at 80 °C for 18 h, cooled down to room temperature and the nanobeads recovered by magnetic decantation. After washing with water, methanol and acetone (5 x 5 mL each), the particles were dried in vacuum. 105 mg of Co/C-Cyclen-Zn nanoparticles **13** could be obtained.
IR (ν/cm^{-1}): 2922, 2854, 1668, 1600, 1505, 1381, 1215, 1014, 963, 833; **elemental microanalysis** (%): C, 8.43; H, 0.16; N, 0.47; **ICP-AES**, Zn: 0.38 mmol/g.

6. General procedure for the reversible extraction of riboflavin

In a typical experiment the magnetic nanoparticles (10 mg) were added into a solution of riboflavin in HEPES-buffer (50 mL, c = 10^{-5} mol/l, pH 7.6) and stirred vigorously for 5 min utilizing the intrinsic magnetic properties of the nanomagnets. Then, the nanoparticles were collected by an external magnet. A sample (1 mL) of the solution was filtered and the absorption- and emission-spectrum recorded. Subsequently, the nanoparticles were washed twice with deionized water and the riboflavin was subsequently released by stirring the particles in diluted hydrochloric acid (50 mL, pH 3.6) for 10 min. The particles were again washed twice with water and reused in the next cycle.

7. NMR spectra

^{1}H-NMR (300 MHz, $CDCl_3$)

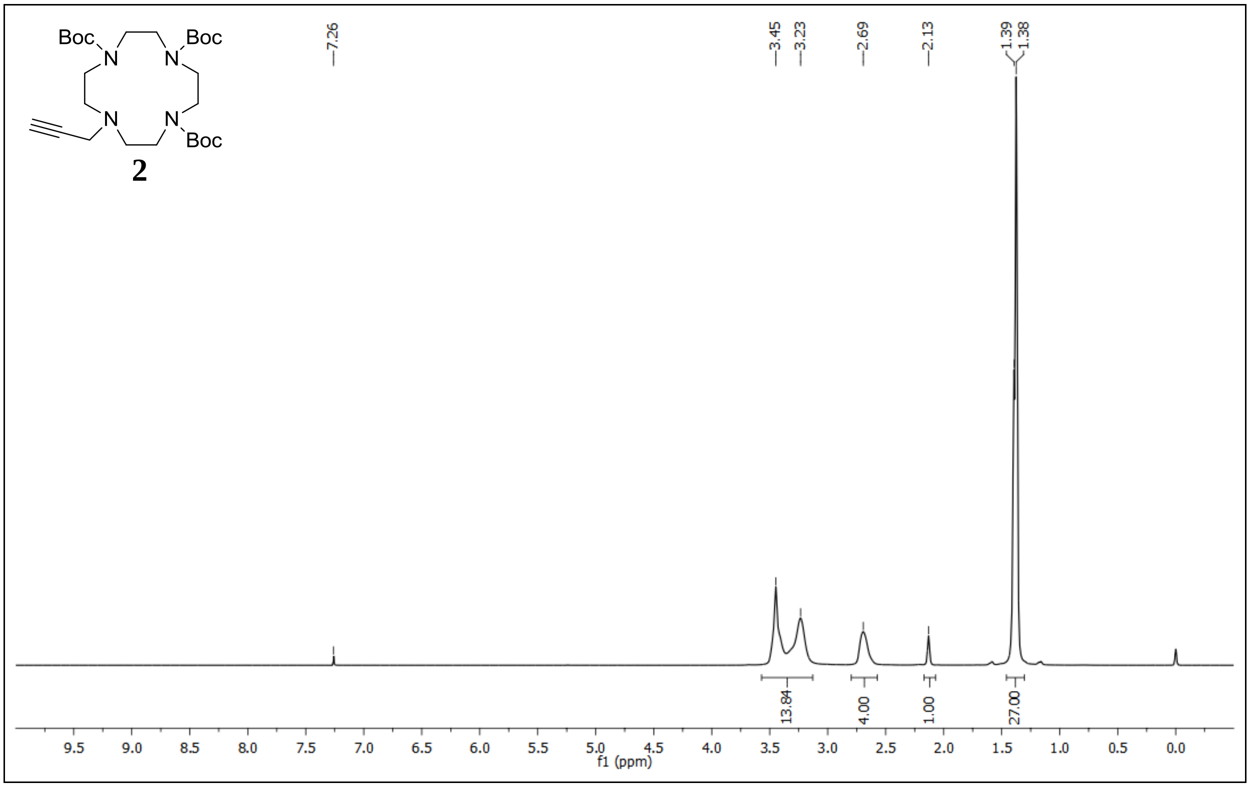

^{13}C-NMR (75.5 MHz, $CDCl_3$)

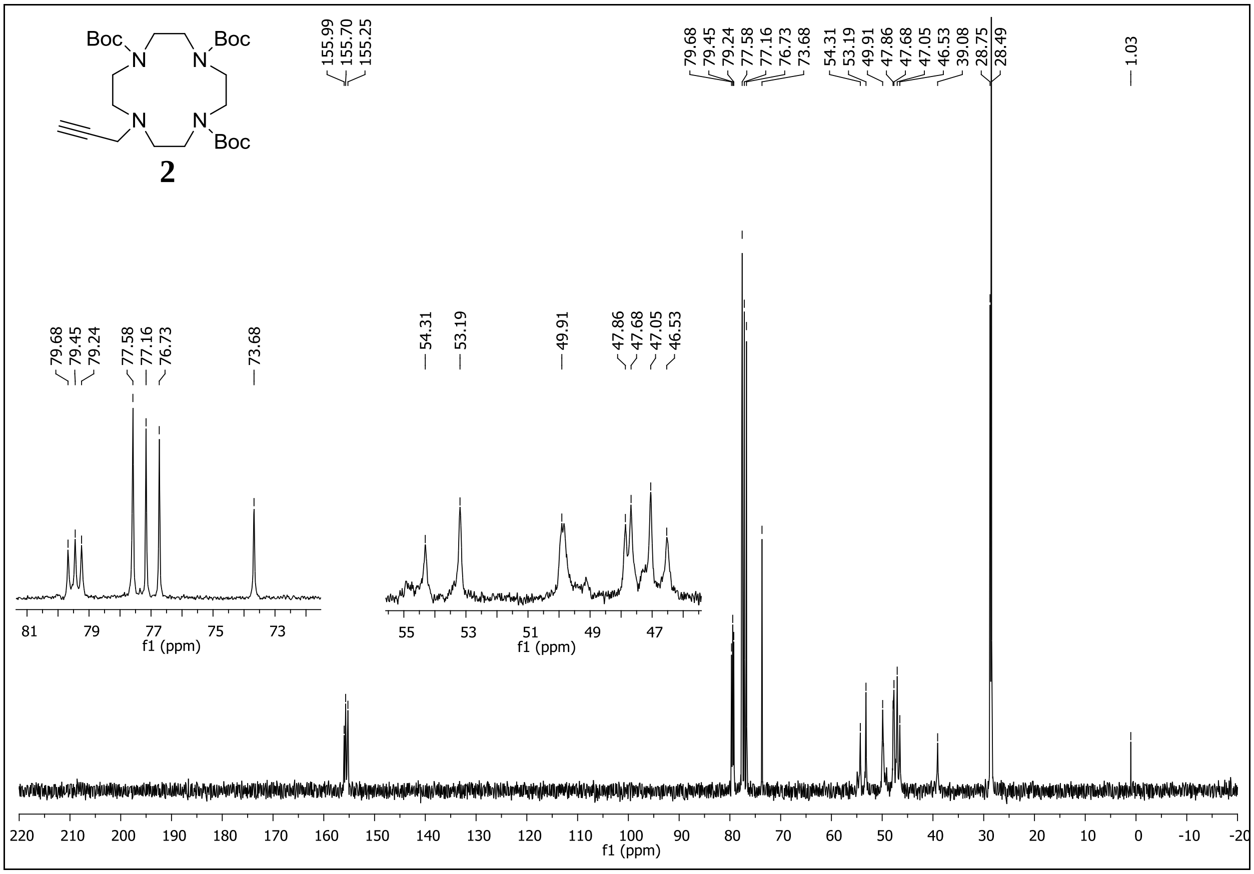

8. IR-ATR spectra

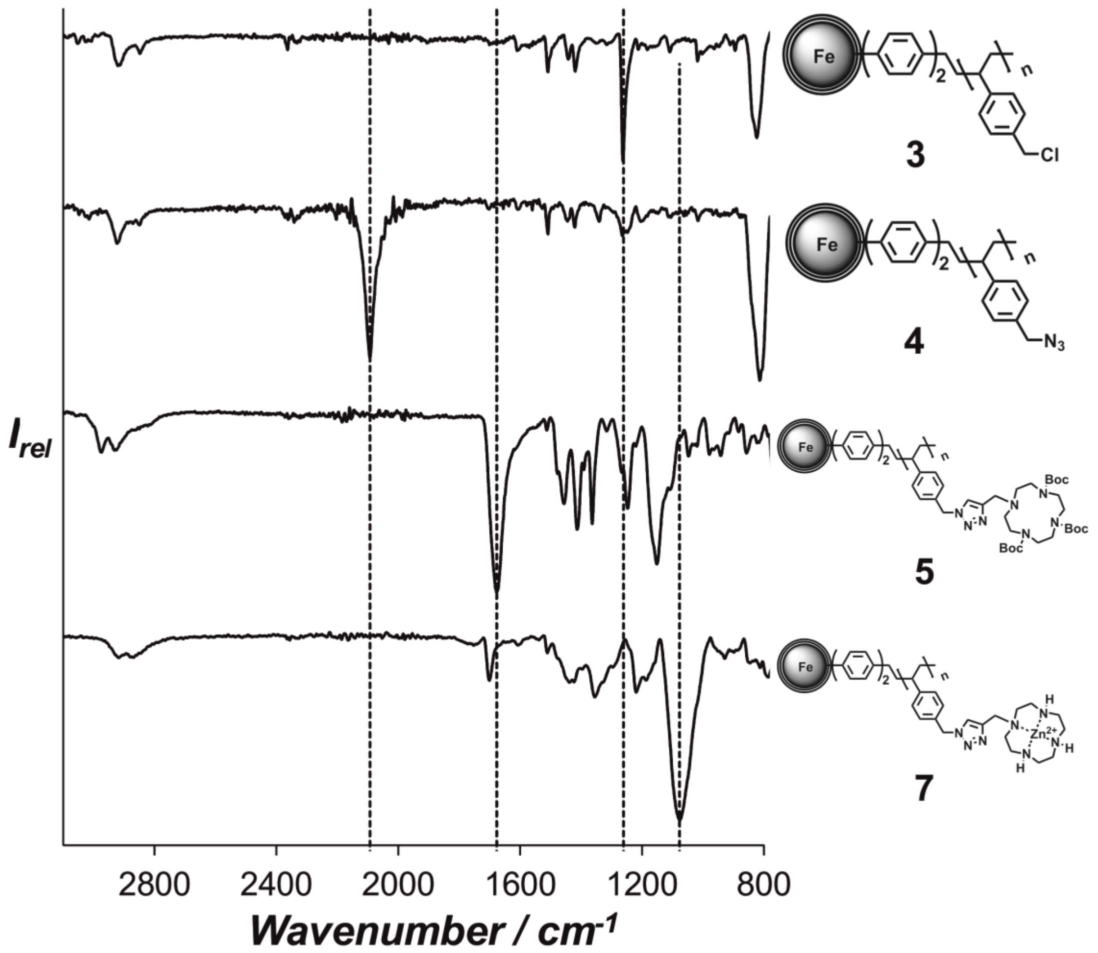

Figure S1. IR Spectra depicting the nucleophilic substitution of the benzyl chloride moieties in **3** with sodium azide and the subsequent attachment of the cyclen ligand followed by complexation with Zn(II).

9. TEM pictures

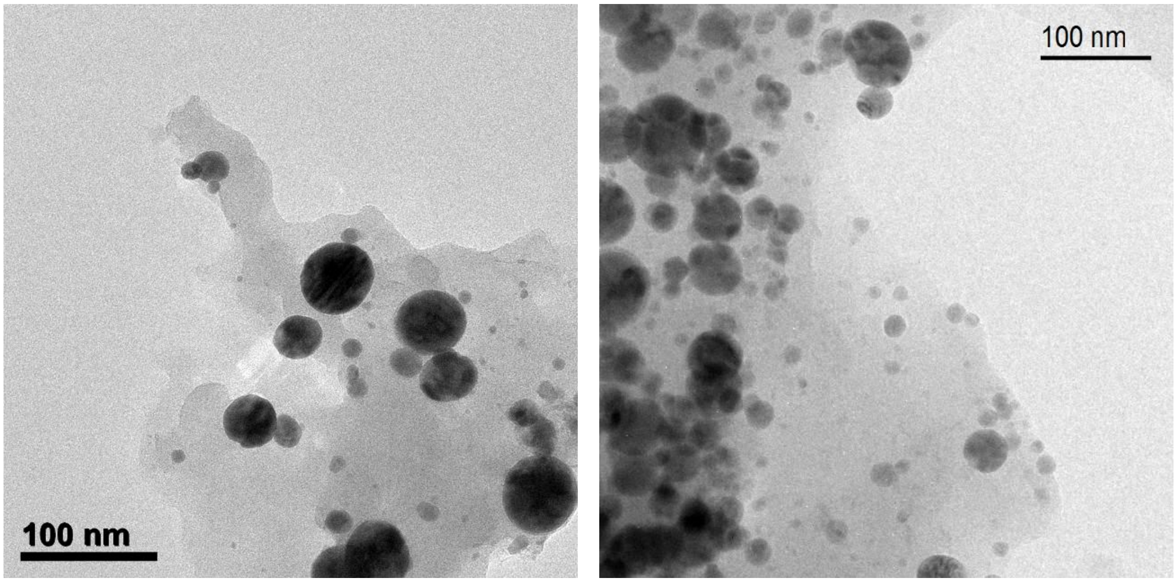

Figure S2. TEM pictures of polymer-encapsulated Fe/C nanoparticles before the functionalization with zinc(II)-cyclen complexes (**3**, left) and after the functionalization (**7**, right). No substantial changes between the pictures are noticeable.

10. Additional UV- and fluorescence-spectra, binding studies

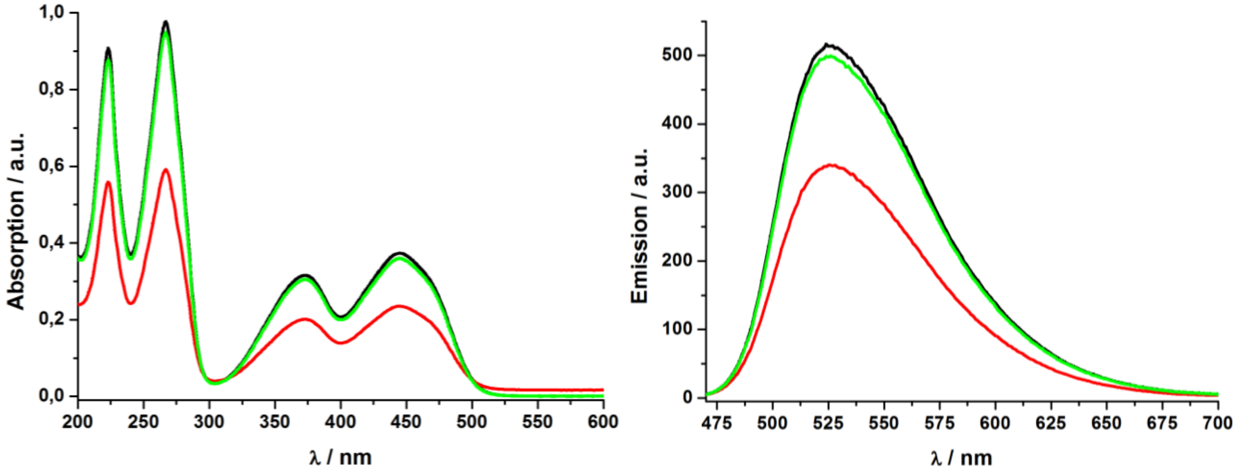

Figure S3. Absorption- (left) and emission- (right) measurements to determine the loading of the functionalized particles. 200 mL of a riboflavin solution (30 µM) and 20 mg of **7** were used. Black line: riboflavin solution (30 µM); red line: solution after treatment with particles; green line: recovery of riboflavin. To recover the riboflavin the particles were washed with 100 ml diluted HCl. From the differences in the intensities a loading of ~ 0.16 mmol/g was calculated.

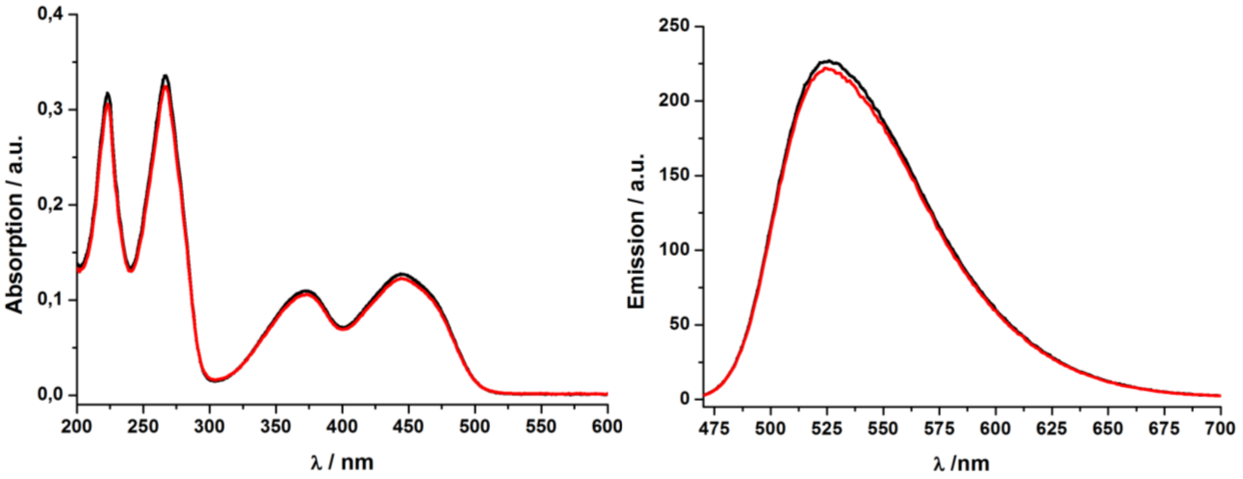

Figure S4. Absorption- (left) and emission- (right) measurement of the polymer coated particles **3** lacking the zinc(II)-cyclen complexes. Black line: riboflavin solution 10 µM; red line: solution after treatment with particles. Only a negligible amount of riboflavin is adsorbed by the particles.

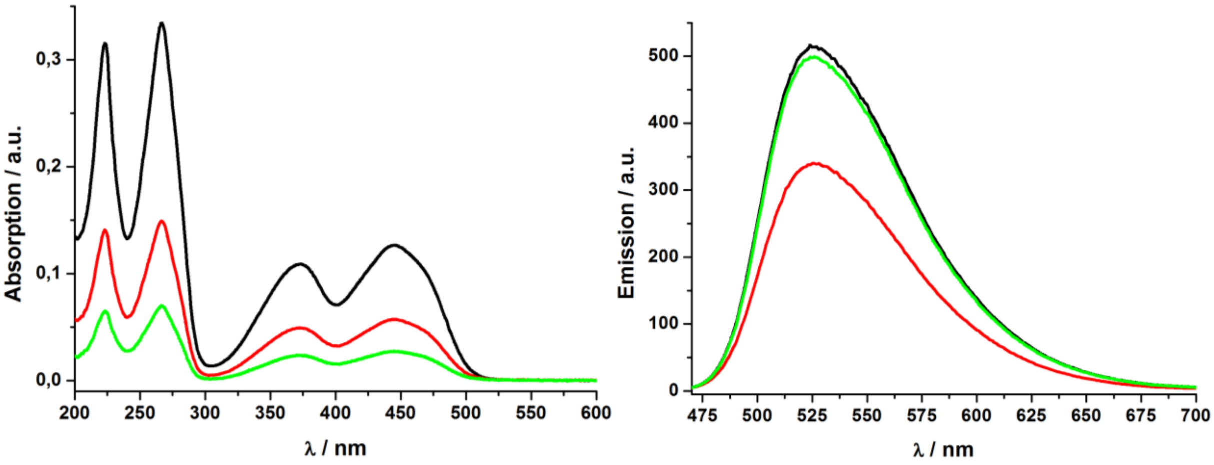

Figure S5. Binding- and release of riboflavin to Zn(II)-cyclen-functionalized Co/C nanoparticles **13**. Absorption spectra (left) as well as emission spectra (right) are shown. Black line: riboflavin solution 10 µM; red line: solution after treatment with particles; green line: recovery of riboflavin.

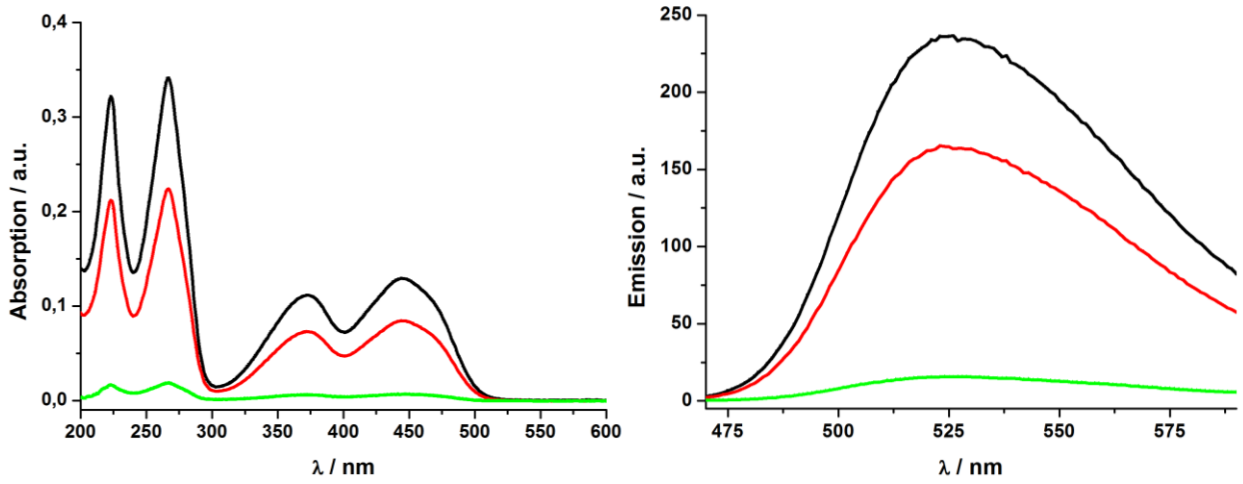

Figure S6. Absorption- (left) and emission- (right) measurement of non-functionalized Co/C particles. Black line: riboflavin solution 10 µM; red line: solution after treatment with particles; green line: recovery of riboflavin. The addition of riboflavin onto the nanoparticle surface is observed to some extent, which cannot be reversed.

11. References

[1] M. Rossier, F. M. Koehler, E. K. Athanassiou, R. N. Grass, B. Aeschlimann, D. Günther, W. J. Stark, *J. Mater. Chem.* **2009**, *19*, 8239–8243.
[2] A. Schätz, M. Zeltner, T. D. Michl, M. Rossier, R. Fuhrer, W. J. Stark, *Chem. Eur. J.* **2011**, *17*, 10566–10573.
[3] a) A. Schätz, R. N. Grass, W. J. Stark, O. Reiser, *Chem.-Eur. J.* **2008**, *14*, 8262–8266; b) A. Schätz, R. N. Grass, Q. Kainz, W. J. Stark, O. Reiser, *Chem. Mater*. **2010**, *22*, 305–310.
[4] Adapted from: Ritter, S.; Dissertation, University of Regensburg, **2007**.
